# *Yersinia pestis* DNA from Skeletal Remains from the 6^th^ Century AD Reveals Insights into Justinianic Plague

**DOI:** 10.1371/journal.ppat.1003349

**Published:** 2013-05-02

**Authors:** Michaela Harbeck, Lisa Seifert, Stephanie Hänsch, David M. Wagner, Dawn Birdsell, Katy L. Parise, Ingrid Wiechmann, Gisela Grupe, Astrid Thomas, Paul Keim, Lothar Zöller, Barbara Bramanti, Julia M. Riehm, Holger C. Scholz

**Affiliations:** 1 State Collection for Anthropology and Palaeoanatomy, Munich, Germany; 2 Department Biology I, Anthropology and Human Genetics, Ludwig Maximilian University of Munich, Martinsried, Germany; 3 Institute for Anthropology, Johannes Gutenberg University, Mainz, Germany; 4 Centre for Ecological and Evolutionary Synthesis (CEES), Department of Biosciences, University of Oslo, Oslo, Norway; 5 Center for Microbial Genetics and Genomics, Northern Arizona University, Flagstaff, Arizona, United States of America; 6 Institute of Palaeoanatomy, Domestication Research and the History of Veterinary Medicine, Department of Veterinary Sciences, Ludwig Maximilian University of Munich, Munich, Germany; 7 Bundeswehr Institute of Microbiology, Munich, Germany; University of Notre Dame, United States of America

## Abstract

*Yersinia pestis*, the etiologic agent of the disease plague, has been implicated in three historical pandemics. These include the third pandemic of the 19^th^ and 20^th^ centuries, during which plague was spread around the world, and the second pandemic of the 14^th^–17^th^ centuries, which included the infamous epidemic known as the Black Death. Previous studies have confirmed that *Y. pestis* caused these two more recent pandemics. However, a highly spirited debate still continues as to whether *Y. pestis* caused the so-called Justinianic Plague of the 6^th^–8^th^ centuries AD. By analyzing ancient DNA in two independent ancient DNA laboratories, we confirmed unambiguously the presence of *Y. pestis* DNA in human skeletal remains from an Early Medieval cemetery. In addition, we narrowed the phylogenetic position of the responsible strain down to major branch 0 on the *Y. pestis* phylogeny, specifically between nodes N03 and N05. Our findings confirm that *Y. pestis* was responsible for the Justinianic Plague, which should end the controversy regarding the etiology of this pandemic. The first genotype of a *Y. pestis* strain that caused the Late Antique plague provides important information about the history of the plague bacillus and suggests that the first pandemic also originated in Asia, similar to the other two plague pandemics.

## Author Summary

Plague is a notorious and fatal human disease caused by the bacterium *Yersinia pestis* that is endemic in many countries around the world. Three of the most devastating pandemics in human history have been associated with plague. The second pandemic originated in central Asia and peaked in Europe between 1348 and 1350 (a period of time known as the Black Death). The third pandemic began in the Yunnan province of China in the mid-1850s and subsequently spread to Africa, the Americas, Australia, Europe, and other parts of Asia. The second and third pandemics are well documented and scientifically proven. However, the first pandemic, which began in the 6^th^ century and is also known as Justinianic Plague, is still a matter of controversy. Recently it has been suggested that Justinian's plague was not caused by *Y. pestis*. We detected *Y. pestis* DNA in samples obtained from multiple 6^th^ century skeletons from Germany. This confirms that Justinianic Plague crossed the Alps and affected local populations there, including current day Bavaria. Furthermore, we used DNA fingerprinting approaches to determine Asia as the likely geographic origin for these strains.

## Introduction

In 541 AD, eight centuries before the Black Death, a deadly infectious disease hit the Byzantine Empire, reaching Constantinople in 542 and North Africa, Italy, Spain, and the French-German border by winter 543 [1]. The so called “Plague of Justinian”, named after the contemporaneous emperor, led to mass mortality in Europe similar to that of the Black Death. It persisted in the territory of the Roman Empire until the middle of the 8^th^ century and likely contributed to its decline, shaping the end of antiquity [1]. Based on historical records, this disease has been diagnosed as bubonic plague although discrepancies between historical sources and the progression of *Y. pestis* infections have led some authors to suppose that the Plague of Justinian was caused by a different pathogen (as discussed in [2]). This vivacious discussion was recently reinforced by an ancient DNA study of the second pandemic that also questioned whether *Y. pestis* was truly the causative agent of the first pandemic [3], [4].

**Figure 1:**
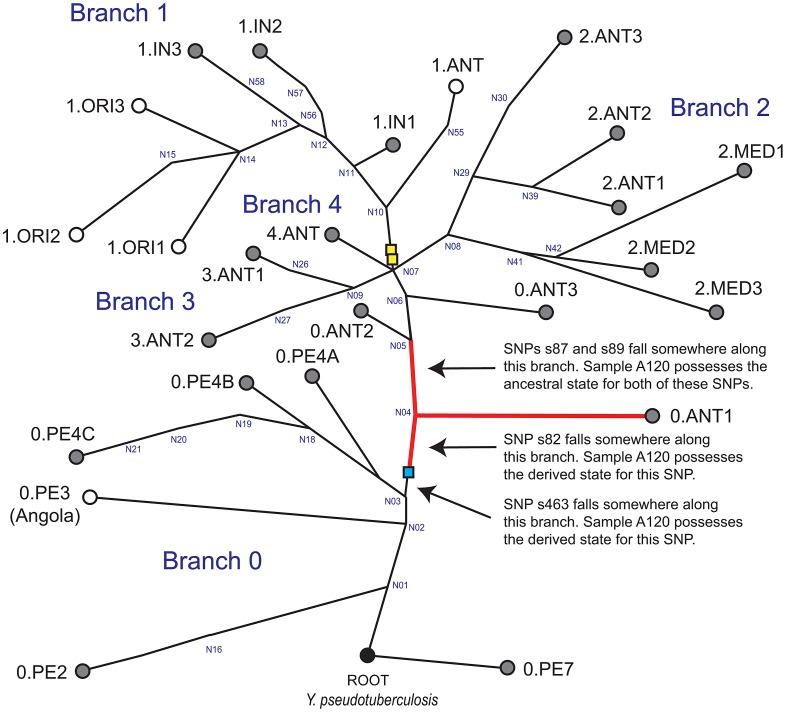
Global phylogeny for *Y.* *pestis*. This global phylogeny for *Y. pestis* is based upon figures 1A and S3B in Cui et al. [11]. It includes four major branches (0–4) and is rooted with *Y. pseudotuberculosis*, the ancestor of *Y. pestis* [28]. The identities of many of the major nodes defined by Cui et al. [11] are presented in blue text. Circles represent specific populations; populations highlighted in gray have, to date, only been found in Asia [10], [11]. Note that the location where strain Angola was isolated, which is the sole representative of population 0.PE3, is unknown. The phylogenetic position of Mongolian strain MNG 2972 is indicated with the blue box (see text). Five previously identified [10], [11] key SNPs were utilized in the current study: s545, which occurs along the branch between nodes N06 and N07 (not shown); s87 and s89; which occur along the branch between N04 and N05; s82, which occurs along the branch between the branching point of strain MNG 2972 and N04; and s463, which occurs along the branch between the branching point of strain MNG 2972 and N03. It is known that these SNPs occur along these specific branches but the exact position and order of these SNPs along each branch is unknown. Sample A120 possesses ancestral states for SNPs s87, s89, and s545; and derived states for SNPs s82 and s463. Thus, the position of sample A120 in this phylogeny is along the branch between the branching point of strain MNG 2972 and N04, along branch N04-N05, along the branch from N04 to 0.ANT1 (red branches), or along one of the sub-branches within 0.ANT.1 (not shown). The phylogenetic positions of strains from the second pandemic [3], [12] are indicated with the yellow boxes according to Cui et al. [11]. The basal node for the 1.ORI group, which caused the third pandemic, is N14 [11].

Western scientists have traditionally subdivided *Y. pestis* strains into three biovars: Antiqua, Medievalis, and Orientalis; depending on their abilities to ferment glycerol and reduce nitrate [5]. However, this system ignores many other *Y. pestis* biovars that have been designated and described by other scientists [see 6,7,8]. Biovars, which are based upon phenotypic properties, do not always correspond directly to specific molecular groups because the same phenotype can result from different mutations [9]. As a result, it has been suggested that groupings within *Y. pestis*, or assignment of unknown strains to specific populations should be based upon molecular signatures and not phenotypes [9]. Fortunately, the recent construction of highly-accurate rooted global phylogenetic trees for *Y. pestis* [10], [11] (reproduced in Figure 1) have facilitated the assignment of isolates to distinct populations. The most recent global phylogeny is based upon single nucleotide polymorphisms (SNPs) identified from the genomes of 133 global strains [11]. All clones that caused the third pandemic belong to populations assigned to the molecular group 1.ORI [10], [11]; the basal node for this group is N14 (Figure 1).

Two recent studies [3], [12] have queried key SNPs in DNA samples obtained from victims of the second pandemic (14^th^ century AD), facilitating the phylogenetic placement of these samples in the most recent global phylogeny [11]. These samples are along the branch between nodes N07 and N10 (Figure 1) close to the “big bang” polytomy at node N07, where major branches 1–4 split from major branch 0 [11]. Specifically, ancient *Y. pestis* DNA samples from two of these studies [3], [12], which were obtained in England and France, are along branch N07-N10 – just one SNP away from the polytomy at N07 [11]. An additional sample from one of these studies [12], which was obtained in the Netherlands, occurs farther along this same branch – three SNPs away from the polytomy at N07 [11].

Only a few previous studies [13]–[15] have described the isolation of *Y. pestis* DNA from victims of the Late Antique pandemic and only one work group [13], [14] attempted to genotype the samples, assigning them to biovar Orientalis, which is now also designated molecular group 1.ORI [9]. However, the authenticity of these results has been questioned repeatedly because current stringent ancient DNA anticontamination protocols (e.g. independent replication) were not utilized [16], [17]. In addition, the robustness of the genotyping approach utilized in one of these studies [13] has been questioned [18]. Finally, it has been suggested [12], [19] that the resulting phylogenetic assignment (i.e. membership in the 1.ORI group) could not have existed at the time of the Justinianic Plague. Indeed, it seems impossible that isolates from the 1.ORI group caused the first pandemic as this group likely evolved only over the last ∼200–210 years [10], [11].

Against this background, we analyzed and genotyped new material from putative Justinian plague victims dated to the 6^th^ century A.D. from an Early Medieval graveyard in Bavaria, Germany. This cemetery, called Aschheim, contained 438 individuals in total and is characterized by a striking number of double and multiple burials clustering in the second half of the sixth century [20]. In an earlier study [15], we reported isolation of *Y. pestis* DNA from two individuals from Aschheim. However, this previous study failed to utilize all of the contamination controls and authentication of results that has been recommended for studies that describe the detection of pathogen DNA in human remains from archeological sites [12], [21].

In this current study we utilized these more stringent approaches and our results confirm that *Y. pestis* was indeed responsible for the Justinianic Plague. More importantly, we were able to genotype the *Y. pestis* DNA present in samples from one individual using five key SNPs from the recent global *Y. pestis* phylogenies [10], [11]. The genotyping results confirm that the *Y. pestis* strain from the Ascheim victim is more basal on the global phylogeny than the *Y. pestis* populations that caused the Black Death and the third pandemic (Figure 1).

## Results

### Screening for *Y. pestis* specific DNA

Assuming that plague victims might have been buried together, we collected teeth from 19 individuals originating from 12 multiple burials from the 6^th^ century at Aschheim (Table 1). All samples were tested for *Y. pestis* specific DNA in a newly built specialized aDNA laboratory in Munich using both quantitative Real-Time PCR (qPCR) and a conventional PCR approach; these approaches targeted a 70 nt portion and a 133 nt portion of the *Y. pestis*-specific *plasminogen activator gene* (*pla*), respectively. This gene, which is located on the multi-copy plasmid pPst that is specific to *Y. pestis*, has been used in several previous studies to test samples from plague skeletons dating to the time of the Black Death [e.g. 12], [22].

**Table 1:** Individuals from the Early Medieval Cemetery Aschheim-Bajuwarenring (Germany) that were analyzed in this study and corresponding results of screening for a portion of the *Y. pestis* specific *plasminogen activator gene* (*pla*).

				*pla*-screening results		
Multiple burial	Number of individuals	Estimated age	Individual	70 nt approach (Munich) [maximal *pla* copy number]^3^	133 nt approach (Munich)^3^	143 nt approach (Mainz)^3^
**I**	3	530–570^1^	A49	neg.	neg.	-
**II**	5	580–600^1^	A56	neg.	neg.	-
		431–544^2^	A58	pos. [7]	neg.	neg.
			A59	neg.	neg.	-
			A60	neg.	neg.	-
**III**	2	530–570^1^	A66	pos. [1]	neg.	-
**IV**	2	570–630^1^	A72	neg.	neg.	-
**V**	5	443–566^2^	A76	pos. [3]	neg.	neg.
		530–570^1^	A77	pos. [1]	neg.	-
			A82	pos. [1]	pos.	-
**VI**	3	590–630^1^	A105	pos. [6]	neg.	neg.
**VII**	3	525–550^1^	A119	neg.	neg.	-
		435–631^2^	A120	pos. [314]	pos.	pos.
**VIII**	2	530–570^1^	A166	pos. [1]	neg.	-
			A167	neg.	neg.	-
**IX**	2	590–630^1^	A197	neg.	neg.	-
**X**	2	590–630^1^	A205	neg.	neg.	-
**XI**	2	530–570^1^	A278	neg.	neg.	-
**XII**	2	600–680^1^	A295	neg.	neg.	-

1 estimated age by archaeological evidence [20] for the multiple burial,

2 estimated age by radiocarbon dating determined for the particular individual (cal 2 sigma),

3 neg = no amplicon, pos = amplification results, - = not tested.

Using qPCR, we repeatedly obtained a specific *pla* amplification fragment from samples obtained from eight individuals although, with the exception of sample A120, the target copy number was extremely low in most of the analyzed DNA extracts (Table 1). In addition, via conventional PCR we repeatedly obtained a longer *pla* amplification fragment from samples from two of these individuals (A82 and A120; Table 1). These amplicons contained *pla* sequences (GenBank accession number KC170159) that were 100% identical to the type strain CO92.

Concurrently, four samples obtained from intact teeth from four different individuals were independently analyzed in a second DNA laboratory (Mainz; Table 1). This analysis involved amplification of a 148 nt *pla* fragment by conventional PCR [12]. Only one of the four samples (from individual A120) produced an amplicon (Table 1). The observable differences in *pla* amplification success across the three PCR approaches utilized in this study (Table 1) are likely a function of the target PCR amplicon sizes. In agreement with typical ancient DNA behavior [23], our amplification success decreased with increasing target length (Table 1).

### Genotyping analysis

We attempted to genotype all of the positive samples. However, likely due to differences in DNA preservation among the samples we were only able to gain reproducible results from samples from one individual, A120 (Table 2). Note that this was the only individual that was found to be *Y. pestis*-positive with all three PCR approaches (Table 1). We queried multiple samples from individual A120 with assays targeting five key SNPs from the most recent global phylogenies for *Y. pestis* [10], [11] and determined whether these samples possessed the ancestral or derived states for these five SNPs (Table 2). These five SNPs occur along specific branches in the *Y. pestis* phylogeny: s545 occurs along the branch between nodes N06 and N07; s87 and s89 occur along the branch between N04 and N05, s82 occurs along the branch between the phylogenetic branching point of Mongolian strain MNG 2972 (see below) and N04, and s463 occurs along the branch between the phylogenetic branching point of strain MNG 2972 and N03 (Figure 1). In the Munich aDNA laboratory we determined that *Y. pestis* DNA samples obtained from individual A120 possess ancestral states for SNPs s545, s87, and s89; and derived states for SNPs s82 and s463 (Table 2). In the second aDNA laboratory (Mainz) we confirmed these results for s82 and s87 (Table 2); assays for the other SNPs were not utilized in this laboratory. Partial alignments of selected SNP regions of sample A120 in comparison to the reference sequences of *Y. pestis* type strain CO92 and strain 91001 (var microtus) are shown in Table 3. In all cases, extraction and PCR negative controls never produced an amplicon when tested with *Y. pestis* specific primers. These results indicate that the phylogenetic position of sample A120 in the global *Y. pestis* phylogeny is along the branch between the phylogenetic branching point of strain MNG 2972 and N04, along branch N04-N05, along the branch from N04 to 0.ANT1, or along one of the sub-branches within 0.ANT.1 (Figure 1).

**Table 2:** Results of molecular assays carried out on samples from individual A120 in two independent aDNA laboratories.

aDNA laboratory	*pla* qPCR	*pla*	s545 qPCR	s87 qPCR	s87	s89	s82	s463
**Munich**								
**1^st^ tooth, 1^st^ extract**	pos	pos	-	anc	neg	-	-	-
**2^nd^ tooth, 1^st^ extract**	pos	pos	anc	anc	anc	-	der	-
**2^nd^ tooth, 2^nd^ extract**	pos	-	anc	-	anc	anc	der	der
**2^nd^ tooth, 3^rd^ extract**	pos	-	-	-	-	anc	-	der
**Mainz**								
**3^rd^ tooth, 1^st^ extract**	-	pos	-	-	anc	-	der	-

Abbreviations: anc: ancestral, der: derived, pos: positive, *pla*: plasminogen activator gene, -: not tested, neg: no amplicon.

**Table 3:** Partial (Munich) and total (Mainz) alignment of amplified consensus sequences regarding several SNPs.

	Source	Position	Sequences	Position
s463	Y.p. CO92 (AL590842.1)	373625	CGCCGCCGCTGGATCAGCATCCAA**T**GGCGGATAATATGATAGACCACTAA	373674
	A120, Munich (KC170160)		CGCCGCCGCTGGATCAGCATCCAA**T**GGCGGATAATATGATAGACCACTAA	
	Y.p. 91001 (AE017042.1)	553941	CGCCGCCGCTGGATCAGCATCCAA**C**GGCGGATAATATGATAGACCACTAA	553990
			************************.*************************	
s82	Y.p. CO92 (AL590842.1)	3639849	TATCTTCTTCCGCGTTATCCAGGG**T**CTGGTCGCTGGGCCATTGATCCCA	3639897
	A120, Munich (KC170161)		TATCTTCTTCCGCGTTATCCAGGG**T**CTGGTCGCTGGGCCATTGATCCCA	
	A120, Mainz		TTCCGCGTTATCCAGGG**T**CTGGTCGCYGAACCATTGATCCCA	
	Y.p. 91001 (AE017042.1)	718460	TATCTTCTTCCGCGTTATCCAGGG**G**CTGGTCGCTGGGCCATTGATCCCA	718412
			************************.************************	
s89	Y.p. CO92 (AL590842.1)	3210077	TGAACGACGGAAATAGTTCATCAG**A**TAGCGTTTGTAAGAATCTGACAGGT	3210126
	Individual A120 (KC170163)		TGAACGACGGAAATAGTTCATCAG**G**TAGCGTTTGTAAGAATCTGACAGGT	
	Y.p. 91001 (AE017042.1)	3041058	TGAACGACGGAAATAGTTCATCAG**G**TAGCGTTTGTAAGAATCTGACAGGT	3041107
			************************.*************************	
s87	Y.p. CO92 (AL590842.1)	2721852	CTATCATGATATTGGTTGCGTGGG**G**CTAAACGCATTAGCAGAGGCGGGTTACGAT	2721798
	A120, Munich (KC170162)		CTATCATGAtATtGGTTgCGTgGG**T**CTAAACGCATTAGCAGAGGCGGGTTACGAT	
	A120, Mainz		GATATTGGTTGCGTGGG**T**CTAAACGCATTAGCAGAGGCGGGTTACGAT	
	Y.p. 91001 (AE017042.1)	2458381	CTATCATGATATTGGTTGCGTGGG**T**CTAAACGCATTAGCAGAGGCGGGTTACGAT	2458327
			************************.************************	

DNA sequences longer than 50 nt are deposited in GenBank (accession numbers are given), SNP positions are indicated by bold letters and a dot in the bottom line. The Y in s82 indicates that either a C or a T was observed at this position, a common artifact attributable to DNA degradation [23], whereas lower case letters indicate poor quality nucleotides.

## Discussion

Our analyses conducted in two separate aDNA laboratories independently confirmed our previous results [15] that some humans buried in the 6^th^ century Ascheim cemetery were infected with *Y. pestis*. These findings confirm that *Y. pestis* was the causative agent of the Justinianic Plague and should end the controversy over the etiological agent of the first plague pandemic. This outcome is contrary to a recent study [3] that questioned whether *Y. pestis* was indeed the causative agent of the first pandemic based upon the assumption that only strains from major branches one and two are pathogenic to humans, which they estimated to have emerged only in the 13^th^ century AD. However, Cui et al. [11] recently determined that most *Y. pestis* lineages are capable of causing human plague and suggested that this capability has been present since *Y. pestis* evolved from its *Y. pseudotuberculosis* ancestor approximately 1,500–6,400 years ago. Thus, they concluded that *Y. pestis* strains pathogenic to humans already existed long before the beginning of the first pandemic.

Another important issue resolved by our study concerns the geographic origin of the Plague of Justinian. The phylogenetic position of our *Y. pestis* samples from the first pandemic (Figure 1) suggests all three plague pandemics were caused by *Y. pestis* strains that originated in Asia. Two recent studies placed the origin of the 1.ORI strains that caused the first pandemic in China [10], [11], and recent phylogenetic placement of samples from the second pandemic [3], [12] near extant strains from China [11] (Figure 1) suggests that strains that caused the second pandemic also originated in this region. The only extant *Y. pestis* strains assigned to the same portion of the global phylogeny (Figure 1) as our Justinian samples from individual A120 are members of group 0.ANT1, which has only been reported from western China [10], [11], and strains from Mongolia [8], such as MNG 2972 (Figure 1). Although multiple historical sources have pointed to an African origin for the Justinian Plague [1], [5], [24], including speculations based on genealogies of *Y. pestis* [11], they have not discussed the original sources of where the bacteria arose. Our results document that those original sources were in Asia.

Cui et al. [11] recently raised the possibility that the Angola strain (sole representative of group 0.PE3; Figure 1) might have spread from Africa to all of Europe and been involved in the first pandemic. They based this hypothesis on several points. First, the Angola strain contains more SNPs than any other known strain of *Y. pestis*, which is consistent with a history of involvement in epidemic waves. Second, their 95% confidence intervals for the age estimates of the nodes that flank Angola (0.PE3) in the global phylogeny, nodes N01 and N03 (Figure 1), are 2,775 BC – 590 AD and 932 BC – 806 AD, respectively, which overlap with the 541 AD date given for the beginning of the first pandemic. Third, they assume that the strain named Angola was actually isolated in Africa in the country of Angola. We do not dispute their first two points. However, we know of no published studies that describe the original isolation of strain Angola making its origins apocryphal. Additional contemporary Angola-like isolates would add insights into this single unique strain type. Although it remains possible that Angola-like strains (ancestors), regardless of its geographic origin, may have been involved in the first pandemic, this remains just a hypothesis until additional samples from the first pandemic are genotyped and found to be closely-related to the Angola strain.

Multiple independent age estimates for our samples are consistent with the timing of the first pandemic. The duration of occupancy of the row burial cemetery at Aschheim-Bajuwarenring has been determined by strong archaeological evidence to range from approximately 500–700 AD [20]. Radiocarbon dating, which has been carried out on three individuals analyzed in this study, including A120 (Table 1), is consistent with this range. Finally, the phylogenetic position of our samples on the global *Y. pestis* phylogeny is on main branch 0 between nodes N03 and N05, with node N04 occurring in between (Figure 1). In their Figure S8, Cui et al [11] provide the 95% confidence intervals for the age estimates for these three nodes. The date given for the beginning of the first pandemic, 541 AD, overlaps with the confidence intervals for nodes N03 and N04, although not with the confidence intervals for N05. Collectively, these various age estimates for our samples provide convincing evidence that they are of the correct age to have been involved in the first plague pandemic.

Our results also provide new stimulus to the discussion about simultaneous multiple inhumations in Europe during the Early Medieval period [25], [26]. It is often presumed that only mass graves are suggestive of a highly infectious disease [27], whereas our results indicate that epidemics can also be indicated by a clustering of simultaneous inhumations involving only two or three individuals (Table 1). This observation may help to identify additional potential victims of the Justinianic Plague. Genetic studies of additional skeletal remains from other plague sites in different geographic regions would not only enhance our knowledge regarding the evolution of the pathogen, but also improve our understanding of the epidemics and spread of the Justinianic Plague. In addition, as there is no known historical source indicating that the Justinianic Plague reached current day Bavaria, our results provide the only evidence that the disease crossed the Alps and affected local populations there [1].

## Materials and Methods

### Material

The burial date of the individuals tested for *Y. pestis* in this study were previously estimated by archaeological methods [20] to fall in a range from 525 to 680 AD (Table 1). To confirm this, we carried out radiocarbon dating on three samples. For individual A58, calibration indicated cal. 431–544 AD (95.4% probability) as the most likely range. Individual A76 from a second burial pit was dated to cal. 443–566 AD (95.4% probability), and individual A120 from a third burial pit was dated to cal. 435–631 AD. (95.4% probability).

From all 19 individuals (Table 1) two or more teeth were taken and analyzed at the aDNA laboratory in Munich. For four individuals (A58, A76, A105, and A120), another intact tooth was sent directly to a second aDNA laboratory in Mainz where they were analyzed independently and blindly.

### Sample preparation and DNA extraction

In Munich the pre-PCR DNA analyses, including the decontamination procedure, DNA extraction, and assembly of the reactions for PCR amplification; were carried out in the new aDNA laboratories at the ArchaeoBioCenter (Ludwig-Maximillians-University, Munich). This aDNA laboratory is located several kilometers from the laboratory used for the post PCR analyses, which included the actual amplification process and sequencing; the post PCR laboratory is situated at the Bundeswehr Institute of Microbiology in Munich. Movement of samples between the laboratories was always unidirectional: from the aDNA laboratories to the post PCR laboratory. The pre-PCR laboratories are dedicated solely to aDNA analysis and have specialized equipment, such as airlocks, HEPA filtered air, positive air pressure, and UV air flow cleaner. In addition, extensive cleaning protocols using bleach and UV irradiation are in place. All possible further methodological precautions were also taken, such as mock extractions, PCR blanks, and independent replications of extractions and amplifications.

In the first step, samples were subjected to decontamination procedures consisting of cleaning the outer surface with a 1% NaOCl solution and exposure to 15 min of UV irradiation on each side, with subsequent powdering using a ZrO_2_-coated mill. DNA extraction in Munich was performed as described previously [15] on powder aliquots of 0.4 g. In Mainz precautions for preventing contamination, pre-treatment of the samples and extraction protocols were as published previously [12].

### Amplification

Every sample analyzed in the Munich laboratory for *Y. pestis* specific DNA (*pla*) was tested at least for three times using the qPCR and conventional PCR approach before considering it negative. Samples that yielded amplification products in any of these PCR reactions were submitted to genotyping assays targeting five key SNPs from the most recent global *Y. pestis* phylogenies [10], [11]: s545 (qPCR approach); s87 (both qPCR and conventional PCR approach); and s82, s89, and s463 (conventional PCR approaches).

For qPCR assays (*pla*), or qPCR SNP endpoint genotyping assays (s87 and s545), we used 1× Platinum Quantitative SuperMix-UDG (Invitrogen), 6 mM MgCl_2_, (Applied Biosystems), 0.4 mg/ml BSA (Ambion/Life Technologies), assay specific primer and probe concentrations (Table 4) (TibMolbiol), and 2.0 to 4.0 µl of template DNA in a final reaction volume of 12 to 24 µl. Primer sequences are listed in Table 4. Cycling conditions comprised an initial step at 50°C for 2 min, an activation step at 95°C for 10 min, 50 cycles at 95°C for 10 sec, and an assay specific annealing temperature for 1 min (Table 4). Final cooling was carried out at 4°C for 30 sec. QPCR assays were carried out on a LightCycler 480 II platform (Roche, Mannheim, Germany). Quantification of *pla*-qPCR assays was possible by determination of the copy numbers per reaction by generating a standard curve using synthetic oligonucleotide constructs. Data analysis was performed using the LightCycler 480 II software version 1.5 (Roche, Mannheim, Germany).

**Table 4:** Design of molecular assays carried out in the Munich laboratories (conc = concentration).

Molecular assay	Primers	Conc (µM)	Probes^1^	Conc (µM)	Annealing temperature (°C)
***pla*** ** qPCR**	fwd_GACTGGGTTCGGGCACATG	0.25	FAM-TGATGAGCACTA+TAT+G+A+GAG-BBQ	0.1	60
	rev_CGGATGTCTTCTCACGGA	0.25			
***pla***	fwd_GACTGGGTTCGGGCACATG	0.2			64–60 (touch down)
	rev_AGACTTTGGCATTAGGTGTG	0.2			
**s545 qPCR**	fwd_ATGCAGACCTGCTTCCTGAAAG	0.9	FAM-CAGCGCAGTCTCCCCG-BBQ	0.4	62
	rev_CCAGATAGTTAAGAAAGCTGTACGTG	0.9	YAK-TCAGCACAGTCTCCCCGACT-BBQ	0.45	
**s87 qPCR**	fwd_AAAATAATCAGGATGTAGAAAAATGAAAG	0.9	FAM-TTAGCCCCACGCAACCAA-BBQ	0.2	62
	rev_CGTAACCCGCCTCTGCTA	0.9	YAK-TTAGACCCACGCAACCAATATCAT-BBQ	0.4	
**s87**	fwd_AAAATAATCAGGATGTAGAAAAATGAAAG	0.2			56
	rev_GGTAAATACCGCCTGAATATCG	0.2			
**s89**	fwd_CTGAATGCGGATTGGCGTC	0.2			58
	rev_GCCAATTGTAGTGATTCACGG	0.2			
**s82**	fwd_GTGCGGCTGTTCTTGTGGTC	0.2			64
	rev_GGCGGATAGTTGTTGAGTAGCAGGC	0.2			
**s463**	fwd_GGCGCGATCAAAGGCAATAC	0.2			60
	rev_CTCACCACCTCACAAGCGCTG	0.2			

1 To minimize the size of PCR amplicons and maximize specificity, we utilized short locked nucleic acid (LNA) probes in our assay. These probes are modified TaqMan probes that were developed by Exiqon (Vedbaek, Denmark) and can be used when high affinity probes as specific as possible are required. The “+” in the sequence of the probe indicates the positions of the LNA labeling.

For conventional PCR assays (*pla*, s82, s87, s89, s463), we used 1× Qiagen Multiplex PCR Master Mix, 0.4 mg/ml BSA, and 2 or 4 µl of DNA in a final volume of 50 µl. Primer sequences are listed in Table 4. The experiments were run on an Eppendorf Mastercycler Pro instrument. Cycling conditions started with an initial activation step at 95°C for 15 min. This was followed by 50 cycles at 94°C for 30 sec, an assay specific annealing temperature (Table2) for 30 sec, and 72°C for 1 min, ending with a final elongation step at 72°C for 10 min. Final cooling was carried out at 8°C until analysis.

Results (*pla* or SNPs) were only considered valid if they could be repeated at least three times from different extracts. Protocols for *pla*, s82, and s87 analysis in the second aDNA lab (Mainz) were carried out as previously published [12].

### Sequencing and alignments

All amplified products were verified by DNA sequencing and BLASTN-analysis.

For the sequencing reactions in Munich we used 1× BigDye terminator v.3.1 Cycle Sequencing Ready reaction Mix (Applied Biosystems), 1 pmol/µl of the respective primers, and 3–5 µl of purified DNA template in a final volume of 10 µl. The reaction was run on a GeneAmp 9700 (Applied Biosystems) instrument, starting with an initial denaturation step for 1 min at 96°C, followed by 25 cycles at 96°C for 10 sec, 50°C for 5 sec and 60°C for 2 mins, and ending with cooling at 4°C until further processing. After purification using the Dye Ex 2.0 Spin Kit (Qiagen) sequences were generated on a Genetic Analyzer 3130 (Applied Biosystems) instrument. Sequences were further analyzed using the program CodonCodeAligner version 4.0. Analyses of the results of the SNPs assays were carried by aligning the amplicons to *Y. pestis* type strain CO92 (AL590842.1), which possessed the derived state for all of the queried SNPs, and *Y. pestis* microtus strain 91001 (AE017042.1), which possessed the ancestral state for all of the queried SNPs. Sequencing in Mainz was carried out as previously described [12]. If long enough, sequences were deposited in GenBank (Accession numbers KC170160-KC170162) and the alignments are shown in Table 3 (only partial sequences are shown for longer sequences).

### Global phylogeny

The global *Y. pestis* phylogeny in Figure 1 is reconstructed from Figures 1A and S3B in Cui et al. [11]. Their phylogeny was constructed using SNPs discovered from the genomes of 133 modern isolates. We have indicated the main branches and molecular groups identified by Cui et al. [11] but not all of their sub-branches and sub-groups. The phylogenetic branching point for Mongolian *Y. pestis* strain MNG 2972 was determined using SNP information provided for this strain in Riehm et al. [8]. Note that, based upon the five SNPs queried in this study, this contemporary Mongolian strain possesses a distinct genotype when compared to the ancient *Y. pestis* DNA samples utilized in this study; the Mongolian strain possesses the ancestral state for s82.

### Accession numbers

The GenBank (http://www.ncbi.nlm.nih.gov) accession numbers for DNA sequences longer than 50 nt determined in this paper are KC170159-KC170163.
